# Changes in Psychological Distress in Five Groups of Welfare State Service Workers over a Nine-Year Period

**DOI:** 10.3390/healthcare10040592

**Published:** 2022-03-22

**Authors:** Amy K. Østertun Geirdal, Per Nerdrum, Per Andreas Høglend, Tore Bonsaksen

**Affiliations:** 1 Department of Social Work, Child Welfare and Social Policy, Faculty of Social Sciences, OsloMet—OsloMetropolitan University, 0170 Oslo, Norway; 2 Senior Centre, OsloMet—Oslo Metropolitan University, 0170 Oslo, Norway; per.nerdrum@gmail.com; 3 Institute of Psychiatry, University of Oslo, 0370 Oslo, Norway; p.a.hoglend@medisin.uio.no; 4 Department of Health and Nursing Science, Faculty of Social and Health Sciences, Inland Norway University of Applied Sciences, 2418 Elverum, Norway; tore.bonsaksen@inn.no; 5 Department of Health, Faculty of Health Studies, VID Specialized University, 4306 Sandnes, Norway

**Keywords:** general health questionnaire, longitudinal study, psychological distress, mental health, health- and social work professions welfare state, service workers

## Abstract

Health and social care workers are exposed to varying degrees of stress in their work, which may be reflected in their trajectories of psychological distress during the education program and the first years in the job. The aim of this study was to add to the knowledge concerned with the long-term development of psychological distress in five groups of welfare state service workers in Norway. The study included 1612 individuals. Psychological distress was measured with General Health Questionnaire-12 at four occasions from the start of the education program to 6 years post-graduation (nine year follow-up period). Results of linear mixed models (LMM) for repeated measures showed that psychological distress changed significantly over time in the social work professional groups. At the start of the education program, the lowest and highest levels of psychological distress were found among the child welfare and social workers, respectively. Six years post-graduation, social workers had become less distressed and child welfare workers had become more distressed. No significant change pattern for psychological distress was found among any of the health care professional groups.

## 1. Introduction

Healthcare and social work professionals, among these are physiotherapists, occupational therapists, social educators, social workers and child welfare workers, provide essential work in the modern Norwegian welfare state. Working in one of these professions can produce challenges that cause emotional exhaustion and psychological distress. Psychological distress has been found to be high among healthcare professionals [1,2,3,4] and social workers [5,6,7,8] in different cultures and contexts. A trend of increased self-reported psychological distress has also been found among students [9]. Over one year, approximately 20% of the Western general population experience psychological distress levels corresponding to a clinically significant burden (case level), and research on students in higher education has reported that 30–40% of these suffer from even higher levels of psychological distress [10,11,12,13]. In Norway, annual student surveys conducted over the last decade have given cause for concern about the development of students’ mental health [14,15,16]. Studies of psychological distress among students have shown variations between different study programs, and it has been assumed that education programs preparing students for caring and treatment professions may evoke more distress than other education programs [16,17,18].

However, researchers have also criticized the notion of higher psychological distress among students compared to other groups. A well-founded critique came from Hunt and Eisenberg [19], who examined ten studies in which students’ mental health was compared with that of same-aged individuals in the general population. These researchers found that the level of, and the increase in, mental health problems in students were like those of their same-aged comparisons. 

Previous Norwegian studies concerned with the period of transition—the time during which students take leave of university and establish themselves as employees in their profession of choice—have found a general decrease in psychological distress during the first years in work, regardless of profession [16,18,20,21]. However, studies have come to different conclusions about the mental health burden in different professional groups. A report from the National Institute of Occupational Health (STAMI) found that 21% of nurses had mental health problems indicating need of treatment, while the corresponding proportion with case-level burden was 11% among teachers [22]. In contrast, Nerdrum et al. [16] found that in comparison to nurses, teachers had higher levels of psychological distress both three and six years after graduation.

Potentially, differences in psychological distress between groups of professionals may have serious long-term consequences. Tufte [23] investigated the risk of disability-related retirement from work in different professional groups. With a particular view to the five professions under scrutiny in the current study, Tufte found that individuals working within these professions had elevated risk of disability-related retirement. In comparison to graduate engineers (reference; 1.0), the risk of disability was 9.9 for social workers, 6.9 for social educators, 6.8 for occupational therapists, 6.7 for child welfare workers and 2.8 for physiotherapists. For employees with lower educational degrees, it was generally concluded that those who worked with people in the service professions had higher risk of disability, compared to those who were not directly involved in the well-being and welfare of individuals [23].

In summary, research has shown high levels of psychological distress among students, while the distress levels appear to be reduced as the students move on to their professional careers. In this study, we present a longitudinal study across nine years concerned with five different professional groups central to the public welfare system in Norway. To our knowledge, such long-term comparative longitudinal studies of psychological distress in these five professional groups have not been previously conducted.

### Study Aim

The aim of this study was to add to the knowledge concerned with the long-term development of psychological distress in five healthcare and social work professional groups in Norway.

## 2. Materials and Methods

### 2.1. Design and Data Collection

A prospective longitudinal design was employed for the study. The data were extracted from the STUD-DATA database [18], constituting self-report data from five professional groups measured at four occasions over a nine-year period. In total, 1612 persons participated in the study. Of these, 448 (27.8%) were social workers, 413 (25.6%) were child welfare workers, 234 (14.5%) were social educators, 198 (12.3%) were occupational therapists, and 319 (19.8%) were physiotherapists. The participants were recruited from six different Norwegian higher education institutions, with the majority of participants from Oslo. The four measurement occasions were at the start of their professional education program (first year students), at the end of their education program (third year students), three years after graduation, and six years after graduation. Thus, the study period included the education period and six years after graduation, i.e., 9 years. The STUD-DATA database consists of 4 panels of participants who responded to 3–4 surveys between 1989 and 2018. Unfortunately, only the second panel responded to the psychological distress measure at all 4 timepoints, i.e., over 9 years, which means that this particular paper is based on data collected between 2002 and 2011.

### 2.2. Measures

#### 2.2.1. Sociodemographic Variables

The demographic variables used in the present study were age in years (continuous), civil status and gender.

#### 2.2.2. The General Health Questionnaire

The 12-item General Health Questionnaire (GHQ-12) is widely used as a self-report measure of psychological distress [24,25]. The measure has been validated in a large number of studies of the general adult population, clinical populations and work populations, as well as in populations of students and young professionals [10,25,26,27,28] and has been translated to and validated in more than 40 languages, among these is Norwegian [29].

Six items of the GHQ-12 are positively phrased (e.g., ‘able to enjoy day-to-day activities’), while six items are phrased negatively (e.g., ‘felt constantly under strain’). For each item, the person indicates the degree to which he or she has experienced the item content during the last fortnight, using four response categories (‘less than usual’, ‘as usual’, ‘more than usual’ or ‘much more than usual’). Items are scored between 0 and 3, where 0 represents the absence of negative stress, 3 represents a high degree of negative stress. As a result, the GHQ-12 scale score range is 0–36, where higher scores indicate more psychological distress.

### 2.3. Statistical Analysis

Continuous variables were described with mean and standard deviation (SD), categorical data with counts and percentages. Group differences regarding age were examined with one-way analysis of variance (ANOVA) and with Chi-square test for gender proportions and marital status. To investigate the reliability of the GHQ-12 in this particular Norwegian sample, we used Cronbach’s alpha to compute internal consistency.

The longitudinal analysis of psychological distress (continuous measure) was performed using linear mixed models (LMM) for repeated measures. Unlike the traditional ANOVA approaches for analyzing repeated measures, LMM allows for estimating trajectories in spite of missing values, provided the participant has valid scores at a minimum of one measurement occasion. It thereby expands on the general linear model to permit the data to exhibit correlated and non-constant variability. LMM models also estimate possible within-individual dependencies by using all available data.

Because repeated measures (level 1) were nested within individuals (level 2), we used a two-level hierarchically nested mixed effects growth model to analyze the longitudinal data. Log transformation of time fitted the data discernibly better than a linear time slope (change in −2 log likelihood). Intercept and logarithmically transformed time were included in both the random and the fixed part of the model. Random intercepts and random slopes were fitted for each person. After the time variable was entered as predictor at Level 1, the education group predictor was entered at Level 2. In this study we compared different combinations of education groups. The reference group in each analysis was coded 0, and the group to be compared with the reference group was coded 1. Type of education was treated as fixed effect only.

The following composite model equation was used to test possible differences in GHQ trajectories between the education groups over the 9-year study period: Y_ij_ = B_0i_ +B_1_ EDUCATION GROUP + B_2_ LNTIME1_ij_ + B_3_ (EDUCATION_i_ × LNTIME1_ij_) + [ζ_0i_ + ζ_1i_ LNTIME_ij_ + ε_ij_]. Y_ij_ is the GHQ score for subject i at time point j. B_0_–B_3_ are the fixed effects and [ζ_0i_ + ζ_1i_ LNTIME_ij_ + ε_ij_] are the random intercept, random time and error term, respectively. The relevant parameters are B_1_, representing the difference in GHQ scores at baseline; B_2,_ representing the slope (amount of change over time) for the education group coded 0; and B_3_, representing the difference in slopes between the education groups being compared. We could detect significant covariance between intercepts and slopes, and an unstructured covariance matrix was selected for the analyses in this study, which yielded the best goodness-of-fit measures in our model. 

Effect sizes (converted to Cohen’s *d*), derived from the *F*-test for mixed effects model, were calculated as $d=2\sqrt{\frac{F}{df}}$, where *F* is the *F*-test statistic for the effect of interest in the repeated model as well as other multilevel designs [30,31].

### 2.4. Ethics

All participants were informed that participation in the study was voluntary and that they could refuse to participate or withdraw from the study at any time. Permission to collect, compute and store the data was approved by The Norwegian Data Inspectorate.

## 3. Results

### 3.1. Participants

A summary of the sample characteristics is displayed in Table 1. In the total sample, the median age of the participants was 22 years (range 18–54 years). The age distribution was similar across professional groups ranging from median 21 to 23 years when starting the study, with the highest median for social educators. There was a predominance of women (*n* = 1340, 83.4%), compared to men (*n* = 266, 16.6%). In comparison to non-responders who were participants in the STUD-DATA dataset but did not respond to the GHQ at any of the measurement occasions, no significant differences occurred with regard to gender proportions. However, the mean age of the sample was somewhat lower than among the non-responders (*M* = 24.11 versus 24.54 years, *p* < 0.05).

No differences were found according to civil status among the groups. The gender proportions differed significantly between the five professional groups, and all groups consisted of more women than men. There was a statistically significant association between gender and professional group; the lowest proportion of women was for physiotherapy, the highest for child welfare workers. 

### 3.2. Developmental Patterns of Psychological Distress

**Figure 1:**
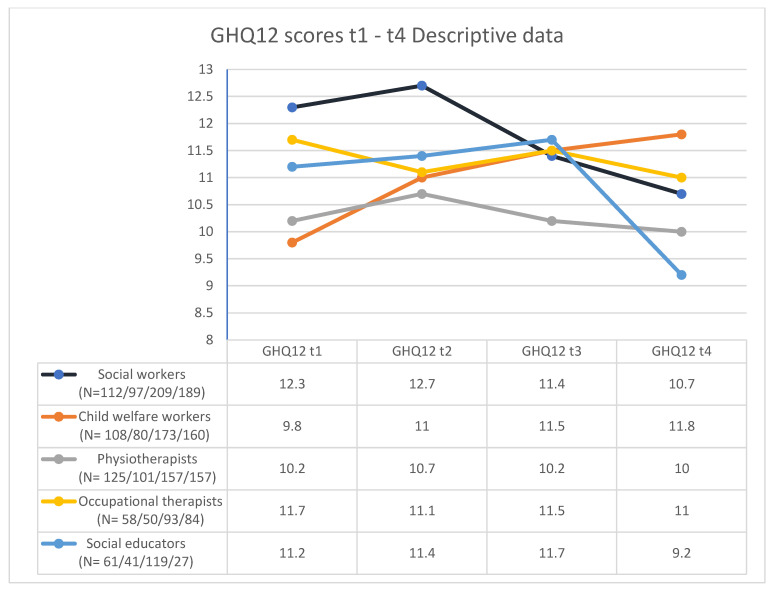
Descriptive data at T1, T2, T3 and T4.

The GHQ-12 had high internal consistency, which indicated good reliability at all measurement times (Cronbach’s alpha = 0.85, 0.87, 0.86 and 0.88). Figure 1 lists the number of participants and descriptive data regarding psychological distress (GHQ scores) for the five education groups at the four time points and illustrates the trajectories over time.

Among social educators, occupational therapists and physiotherapists we detected no significant change over the 9-year time period. On the other hand, psychological distress in social workers and child welfare workers changed significantly over time. Table 2 shows intercept and slopes comparing estimated GHQ trajectories in these two education groups throughout the nine-year study period. B_1_ shows that the difference between the groups at baseline was significant (as also reported in the descriptive analyses). B_2_ shows that the child welfare workers deteriorated significantly over time (effect size = 0.34). B_3_ shows the difference in slopes between the two education groups (effect size = 0.48). While the social workers improved over time, the child welfare workers reported increased psychological distress over the follow-up period., 1.40 − 2.67 = −1.27 (lower scores over time indicates improvement).

## 4. Discussion

At the start of the education program, the lowest and highest levels of psychological distress were found among child welfare and social workers, respectively (Figure 1). However, this situation was reversed during the 9-year period. Social workers became less distressed and child welfare workers became more distressed during this period. On the other hand, no significant changes in psychological distress over time were found among social educators, physiotherapists and occupational therapists.

However, descriptively, as can be seen from Figure 1, the GHQ mean scores showed a slight increase during the education period among all educational groups but occupational therapists. Similarly, all groups except from the child welfare workers showed decreased psychological distress during the first years as an employee. The findings related to the education period mirror the results of some studies. For example, Zivin et al. [32], who conducted a study among college students with two measurement points, found that approximately half of the students suffered from at least one mental health problem. The mixed findings in our study, with both decreasing and increasing psychological distress for different groups, are in line with former studies that have examined psychological distress from education and into the first three years after education in healthcare profession [21] and among other professional than in the particular study [16].

Experiencing stress over time may increase the level of psychological distress. For example, higher levels of job demand have been found to be associated with higher levels of psychological distress in healthcare and social workers and teachers [33], as well as quality of life being negatively affected by psychological distress in these professionals [33]. The respondents in our study represent students and workers in different caring and treatment professions. For people representing these professions, psychological distress may be particularly high for a number of reasons. For example, they may more often witness suffering in others and may feel a strong obligation to help, even in the face of client resistance and system-based obstacles for providing help. Thus, mental health problems in health and social work professionals might be the result of long-term psychological stress during the education program and later in the job.

However, moral distress is a common phenomenon in the daily life of those who work in the social and healthcare professions, which occurs when the professional does not act, or cannot act, on what he or she believes to be morally right in a particular situation because of institutional or internal constraints [34]. Social and healthcare professionals care for people of all ages and in different life circumstances, but often with little fanfare or recognition, and they sometimes perceive their level of competence to be inadequate. In our study, this may seem to affect child welfare workers most. While they had lower scores on psychological distress than all the other student groups in general, and lower than social work students in particular, a plausible explanation for their increased level of psychological distress 6 years past graduation may be the perceived gap between work-related expectations and the realities of daily work. According to Pryce et al. [35], child welfare workers may take in the traumas of the children and families they meet in the line of duty. In addition, they may experience a lot of critique concerned with the decisions they make, both from individuals, groups, society and the media, but with little opportunity to defend or argue in favor of their decisions due to confidentiality and professional ethical considerations. In the health and social service professions, a significant source of stress is often referred to as the practice shock [36,37,38,39], denoting the stressful feelings arising when the reality of professional practice appears to be far from the theoretical descriptions disseminated to students during their education programs. This is likely to contribute to elevated psychological distress. In line with this perspective, Kim [40] reported higher workload, stronger role conflicts and more depersonalization among child welfare workers than among social workers. Similarly, Sprang et al. [41] reported more compassion fatigue and burnout in this group of workers compared to other groups of therapists and social and health care workers. Taken together, our findings support the notion that child welfare workers are among those who experience most strain in their work.

### 4.1. Future Research

According to Harvey et al. [42], several aspects of the work environment, including job demands, level of perceived control, interpersonal support and organizational factors, play an important part in determining the mental health of individual workers. Recently, conflict related to the work–home interaction and job demand were found to be significantly related to psychological distress in several groups of healthcare professionals [20,33]. Future studies may continue to examine mental health in different professional groups and may focus on assessing whether aspects of the work environment that may affect mental health differ systematically between groups. Future studies may also consider individual expectations towards the job and its requirements as another explanation. Another line of future research may investigate whether there are systematic differences with regard to individual factors between groups of students at the time of admission to the study programs, and the degree to which these are maintained throughout the course of the study, at the period of transition to work and during the first years of work. Moreover, some authors have argued a longstanding female predominance in the ‘helping professions’ [43,44,45]. While this notion has been vigorously debated by others [46], our results indicate that there may be reason to study more carefully the male students who come to the healthcare and social work professions, and the factors of importance for sustaining their mental health.

### 4.2. Study Strengths and Limitations

The longitudinal study design with four measurement occasions across a nine-year study period are major strengths of the study. Similarly, the relatively large sample size consisting of individuals representing five professional groups, and the use of modern statistical techniques for analyzing repeated measures, are notable strengths. Limitations of the study are related to participant non-response and attrition, and to a lack of information about the participants’ individual characteristics and about the study and working environments they have been exposed to during the follow-up period.

## 5. Conclusions

The aim of this study was to add to the knowledge concerned with the long-term development of psychological distress in five healthcare and social work professional groups in Norway. The levels of distress did not change significantly over time in any of the three health-care groups, while it showed a significant decrease among social workers and an increase among child welfare workers. It seems plausible that learning about, witnessing, and potentially vicariously experiencing traumatic events imposed on children and their families add substantially to the psychological distress of child welfare workers. Moreover, being morally and legally responsible for implementing effective interventions in often very complex circumstances can be burdensome. However, not all of the variation can be explained adequately with reference to inherent properties of the professional groups and the environments within which they work. On the other hand, the results might be helpful in developing prevention programs for early burnout in the target group.

## Figures and Tables

**Table 1:** Mean age and gender proportions in the total sample and in the five professional groups.

Groups	*n*	Median Age	Female Gender [*n* (%)]
Social workers	448	22.0	384 (85.9)
Child welfare workers	413	22.0	362 (88.1)
Social educators	234	23.0	192 (82.1)
Occupational therapists	198	21.0	169 (86.2)
Physiotherapists	319	22.0	233 (73.3)
Total sample	1612	22.0	1340 (83.4)

**Table 2:** Predictors of GHQ scores in social workers and child welfare workers: Estimates of fixed and random effects.

Parameter	Estimate	Std. Error	df	t	*p*
Intercept (B_0_)	10.01	0.47	257.00	21.48	0.000
Education group (B_1_)	2.67	0.65	262.67	4.11	0.000
LNTime (B_2)_	1.40	0.44	331.93	3.93	0.002
LNTime1xEducation (B_3_)	−2.67	0.61	342.09	−4.37	0.000
Random effects	Estimate	Std. Error		Wald Z	Sig
Residual	18.37	1.24		14.81	0.000
Intercept	8.70	2.71		3.20	0.001
LNTime	7.92	3.64		2.18	0.030
Covariance	−5.01	2.87		−1.7	0.082

Note. Dependent variable: GHQ. Social workers = 1, Child welfare workers = 0.

## Data Availability

The data presented in this study are available on request from the corresponding author by completion of the research. The data is not publicly available due to ongoing publications.

## References

[1] Kabito G.G., Mekonnen T.H. (2020). Psychological distress symptoms among healthcare professionals are significantly influenced by psychosocial work context, Ethiopia: A cross-sectional analysis. PLoS ONE.

[2] Tei-Tominaga M., Nakanishi M. (2018). The influence of supportive and ethical work environments on work-related accidents, injuries, and serious psychological distress among hospital nurses. Int. J. Environ. Res. Public Health.

[3] Aiken L.H., Sloane D.M., Bruyneel L., Heede K.V.D., Sermeus W. (2013). Nurses’ reports of working conditions and hospital quality of care in 12 countries in Europe. Int. J. Nurs. Stud..

[4] Bruschini M., Carli A., Burla F. (2018). Burnout and work-related stress in Italian rehabilitation professionals: A comparison of physiotherapists, speech therapists and occupational therapists. Work.

[5] Kagan M., Itzick M. (2019). Work-related factors associated with psychological distress among social workers. Eur. J. Soc. Work.

[6] Adams R.E., Boscarino J.A., Figley C.R. (2006). Compassion fatigue and psychological distress among social workers: A validation study. Am. J. Orthopsychiatry.

[7] Grant L., Kinman G., Alexander K. (2014). What’s all this talk about emotion? Developing emotional intelligence in social work students. Soc. Work Educ..

[8] Griffiths A., Royse D., Culver K., Piescher K., Zhang Y. (2017). Who stays, who goes, who knows? A state-wide survey of child welfare workers. Child. Youth Serv. Rev..

[9] Knapstad M., Sivertsen B., Knudsen A.K., Smith O.R.F., Aarø L.E., Lønning K.J., Skogen J.C. (2021). Trends in self-reported psychological distress among college and university students from 2010 to 2018. Psychol. Med..

[10] Gorter R., Freeman R., Hammen S., Murtomaa H., Blinkhorn A., Humphris G. (2008). Psychological stress and health in undergraduate dental students: Fifth year outcomes compared with first year baseline results from five European dental schools. Eur. J. Dent. Educ..

[11] Grant P.M., Huh G.A., Perivoliotis D., Stolar N.M., Beck A.T. (2012). Randomized trial to evaluate the efficacy of cognitive therapy for low-functioning patients with schizophrenia. Arch. Gen. Psychiatry.

[12] Henning K., Ey S., Shaw D. (1998). Perfectionism, the impostor phenomenon and psychological adjustment in medical, dental, nursing and pharmacy students. Med. Educ..

[13] Monk E.M. (2004). Student mental health: The case studies. Couns. Psychol. Q..

[14] Nedregård T., Olsen R. (2014). Student’s Health and Wellbeing Study 2014.

[15] Knapstad M., Heradstveit O., Sivertsen B. (2018). Students’ Health and Wellbeing Study 2018.

[16] Nerdrum P., Geirdal A.Ø., Høglend P.A. (2016). Psychological Distress in Norwegian Nurses and Teachers over Nine Years. Prof. Prof..

[17] Dziegielewski S.F., Turnage B., Roest-Marti S. (2004). Addressing stress with social work students: A controlled evaluation. J. Soc. Work Educ..

[18] Nerdrum P., Rustøen T., Helge Rønnestad M. (2009). Psychological Distress among Nursing, Physiotherapy and Occupational Therapy Students: A Longitudinal and Predictive Study. Scand. J. Educ. Res..

[19] Hunt J., Eisenberg D. (2010). Mental Health Problems and Help-Seeking Behavior among College Students. J. Adolesc. Health.

[20] Bonsaksen T., Nerdrum P., Østertun A. (2021). Geirdal, Psychological distress and its associations with psychosocial work environment factors in four professional groups: A cross-sectional study. Nurs. Health Sci..

[21] Nerdrum P., Geirdal A.Ø. (2013). Psychological distress among young Norwegian health professionals. Prof. Prof..

[22] Sterud T. Psychosocial and Organizational Work Environments and Health [Psykososialt og Organisatorisk Arbeidsmiljø og Helse] 2010. https://docplayer.me/1078780-Psykososialt-og-organisatorisk-arbeidsmiljo-og-helse.html.

[23] Tufte P.A. (2013). Risky Professions? Risk of Disability in Professions in Norway. Prof. Prof..

[24] Goldberg D.P., Gater R., Sartorius N., Ustun T.B., Piccinelli M., Gureje O., Rutter C. (1997). The validity of two versions of the GHQ in the WHO study of mental illness in general health care. Psychol. Med..

[25] Goodwin L., Ben-Zion I., Fear N.T., Hotopf M., Stansfeld S.A., Wessely S. (2013). Are reports of psychological stress higher in occupational studies? A systematic review across occupational and population based studies. PLoS ONE.

[26] Adlaf E.M., Gliksman L., Demers A., Ma B.N.-T. (2001). The Prevalence of Elevated Psychological Distress among Canadian Undergraduates: Findings from the 1998 Canadian Campus Survey. J. Am. Coll. Health.

[27] Firth J. (1986). Levels and sources of stress in medical students. Br. Med. J..

[28] Aalto A.M., Elovainio M., Kivimäki M., Uutela A., Pirkola S. (2012). The Beck Depression Inventory and General Health Questionnaire as measures of depression in the general population: A validation study using the Composite International Diagnostic Interview as the gold standard. Psychiatry Res..

[29] Malt U.F., Mogstad T.E., Refnin I.B. (1989). Goldberg’s General Health Questionnaire. Tidsskr. Nor. Laegeforen..

[30] Rosenthal R., Rosnow R.L. (1991). Essentials of Behavioral Research: Methods and Data Analysis.

[31] Verbeke G., Molenberghs G. (2009). Linear Mixed Models for Longitudinal Data.

[32] Zivin K., Eisenberg D., Gollust S.E., Golberstein E. (2009). Persistence of mental health problems and needs in a college student population. J. Affect. Disord..

[33] Geirdal A., Nerdrum P., Bonsaksen T. (2019). The transition from university to work: What happens to mental health? A longitudinal study. BMC Psychol..

[34] Ulrich C.M., Grady C. (2018). Moral Distress in the Health Professions.

[35] Pryce J.G., Shackelford K.K., Pryce D.H. (2007). Secondary Traumatic Stress and the Child Welfare Professional.

[36] Caspersen J. (2013). Professionalism among Novice Teachers: How They Think, Act, Cope and Perceive Knowledge.

[37] Cejda B.D. (1997). An examination of transfer shock in academic disciplines. Community Coll. J. Res. Pract..

[38] Halfer D., Graf E. (2006). Graduate Nurse Perceptions of the Work Experience. Nurs. Econ..

[39] Stokking K., Leenders F., De Jong J., Van Tartwijk J. (2003). From student to teacher: Reducing practice shock and early dropout in the teaching profession. Eur. J. Teach. Educ..

[40] Kim H. (2011). Job conditions, unmet expectations, and burnout in public child welfare workers: How different from other social workers?. Child. Youth Serv. Rev..

[41] Sprang G., Craig C., Clark J. (2011). Secondary traumatic stress and burnout in child welfare workers: A comparative analysis of occupational distress across professional groups. Child Welf..

[42] Harvey S.B., Joyce S., Tan L., Johnson A., Nguyen H., Modini M., Groth M. (2014). Developing a Mentally Healthy Workplace: A Review of the Literature.

[43] Pollard N., Walsh S. (2000). Occupational therapy, gender and mental health: An inclusive perspective. Br. J. Occup. Ther..

[44] Sebrant U. (1999). Being female in a health care hierarchy. Scand. J. Caring Sci..

[45] Bonsaksen T., Kvarsnes H., Dahl M. (2016). Who wants to go to occupational therapy school? Characteristics of Norwegian occupational therapy students. Scand. J. Occup. Ther..

[46] McPhail B.A. (2004). Setting the Record Straight: Social Work Is Not a Female-Dominated Profession. Soc. Work.

